# Retinoic Acid Alleviates Accelerated Atherosclerosis in Diesel Exhaust–Derived PM2.5–Exposed ApoE−/− Mice by Enhancing eNOS–NO Coupling

**DOI:** 10.3390/nu18213680

**Published:** 2026-11-06

**Authors:** Ji-Ye Han, Byung Hyuk Han, Se Hoon Jang, Bori Lee, Young-Mi Lee, Yun Jung Lee

**Affiliations:** 1 Department of Oriental Pharmacy, College of Pharmacy, Wonkwang University, Iksan 54538, Republic of Korea; 2 Wonkwang-Oriental Medicines Research Institute, Wonkwang University, Iksan 54538, Republic of Korea; 3 Hanbang Cardio-Renal Syndrome Research Center, Wonkwang University, 460, Iksan-daero, Iksan 54538, Republic of Korea

**Keywords:** PM2.5, retinoic acid, atherosclerosis, vascular inflammation, BH4

## Abstract

Background: Respiratory exposure to fine particulate matter (PM2.5), including diesel exhaust particles (DEPs), is a major air pollutant associated with pulmonary inflammation and cardiovascular diseases. This study investigated the protective effects and underlying mechanisms of retinoic acid (RA) against PM2.5-induced lung injury and accelerated atherosclerosis. Methods: Differentially expressed genes (DEGs) were identified from lung tissues of five non-smokers and six patients with chronic obstructive pulmonary disease obtained from the Gene Expression Omnibus (GEO) database and analyzed using the L1000CDS^2^, which identified RA as a candidate therapeutic compound. The Comparative Toxicogenomics Database was subsequently used to investigate curated associations between lung and cardiovascular disorders, suggesting the endothelial nitric oxide synthase (eNOS)/nitric oxide (NO) signaling pathway as a candidate mechanism for experimental validation. ApoE−/− mice were exposed to DEP via a nebulization chamber and treated orally with all-trans retinoic acid (1 or 5 mg/kg/day) for 8 weeks. Results: RA significantly reduced C-X-C motif chemokine ligand 1/2 (CXCL1/2) levels in bronchoalveolar lavage fluid and decreased pulmonary expression of Muc5ac, IL-1β, IL-6, and TNF-α. RA also lowered blood pressure, improved serum lipid profiles, and attenuated inflammatory cell infiltration and arterial wall thickening in the thoracic aorta. In HUVECs, RA significantly increased guanosine triphosphate (GTP) cyclohydrolase-1 (GTPCH-1) expression, eNOS phosphorylation, tetrahydrobiopterin (BH4) levels, and the BH4/BH2 ratio. Conclusion: These findings suggest that the activation of the GTPCH-1/BH4/eNOS coupling pathway contributes to the protective effects of RA against DEP-induced cardiovascular injury.

## 1. Introduction

Fine particulate matter (PM2.5) is a major component of air pollution and an important environmental risk factor for human health. Diesel exhaust particles (DEPs), a major source of PM2.5, contain a complex mixture of toxic organic and inorganic compounds and can induce oxidative stress, pulmonary inflammation, and tissue injury following inhalation [1,2]. CXCL1 and CXCL2 may promote the recruitment of inflammatory cells during pulmonary inflammation induced by DEP exposure. Increasing evidence indicates that PM2.5 exposure not only causes pulmonary injury but also promotes systemic inflammation, endothelial dysfunction, and cardiovascular injury [3]. Atherosclerosis is a progressive chronic inflammatory disease of the arterial wall in which endothelial dysfunction plays a critical role in disease initiation and progression. PM2.5 exposure can accelerate atherosclerosis, particularly in the presence of pre-existing cardiovascular risk factors, through pulmonary inflammation, oxidative stress, and subsequent vascular dysfunction [4,5]. DEP exposure has also been shown to aggravate vascular inflammation and endothelial injury, thereby potentially accelerating atherosclerotic progression. However, the molecular mechanisms linking PM-induced pulmonary injury to accelerated atherosclerosis remain incompletely understood, and effective therapeutic strategies for preventing or attenuating PM-induced cardiovascular complications have not yet been sufficiently established.

Recent advances in transcriptomic analysis and computational drug-repurposing approaches provide new opportunities to identify potential therapeutic compounds from disease-associated gene expression profiles. The Gene Expression Omnibus (GEO) is a publicly available repository of gene expression datasets that enables the identification of disease-associated transcriptional alterations [6]. By integrating differentially expressed genes (DEGs) with reference perturbational gene expression signatures, the L1000 Characteristic Direction Signature Search Engine (L1000CDS^2^) can identify chemical perturbations predicted to reverse disease-associated gene expression patterns [7,8]. Therefore, this approach may provide an efficient strategy for screening candidate compounds with potential therapeutic effects on disease-associated biological processes.

Using this approach, retinoic acid (RA) was identified as a promising candidate for further investigation. RA is a biologically active metabolite of vitamin A that regulates diverse physiological processes, including cellular differentiation, inflammation, oxidative stress, and vascular homeostasis [9,10]. Previous studies have suggested that RA exerts protective effects on the cardiovascular system and can modulate endothelial function and inflammatory responses [11]. However, whether RA can protect against PM-induced pulmonary and vascular injury and, in particular, attenuate the acceleration of atherosclerosis induced by DEP exposure remains unclear. As a natural bioactive metabolite, RA was selected as a candidate compound for investigating potential therapeutic effects against PM-induced cardiovascular injury. Tretinoin, also known as all-trans retinoic acid (ATRA), is the biologically active ingredient of RA.

Because pulmonary inflammation and atherosclerosis are closely interconnected through systemic inflammatory and vascular responses, the identification of molecular pathways commonly associated with these disorders may provide insight into the mechanisms underlying PM-induced acceleration of atherosclerosis. In this study, the Comparative Toxicogenomics Database (CTD) was used to explore curated gene–disease associations linking pulmonary and cardiovascular disorders [12,13]. Subsequent functional and pathway analyses suggested the involvement of endothelial nitric oxide synthase (eNOS)/nitric oxide (NO) signaling. The eNOS/NO system is a critical regulator of vascular endothelial homeostasis, and the disruption of eNOS coupling can impair NO production and promote endothelial dysfunction [14,15].

Therefore, the aim of this study was to determine whether DEP exposure exacerbates pulmonary and cardiovascular injury in ApoE−/− mice predisposed to atherosclerosis and to evaluate whether tretinoin treatment can attenuate these effects.

## 2. Materials and Methods

### 2.1. Identification of RA Using L1000CDS^2^

**Figure 1:**
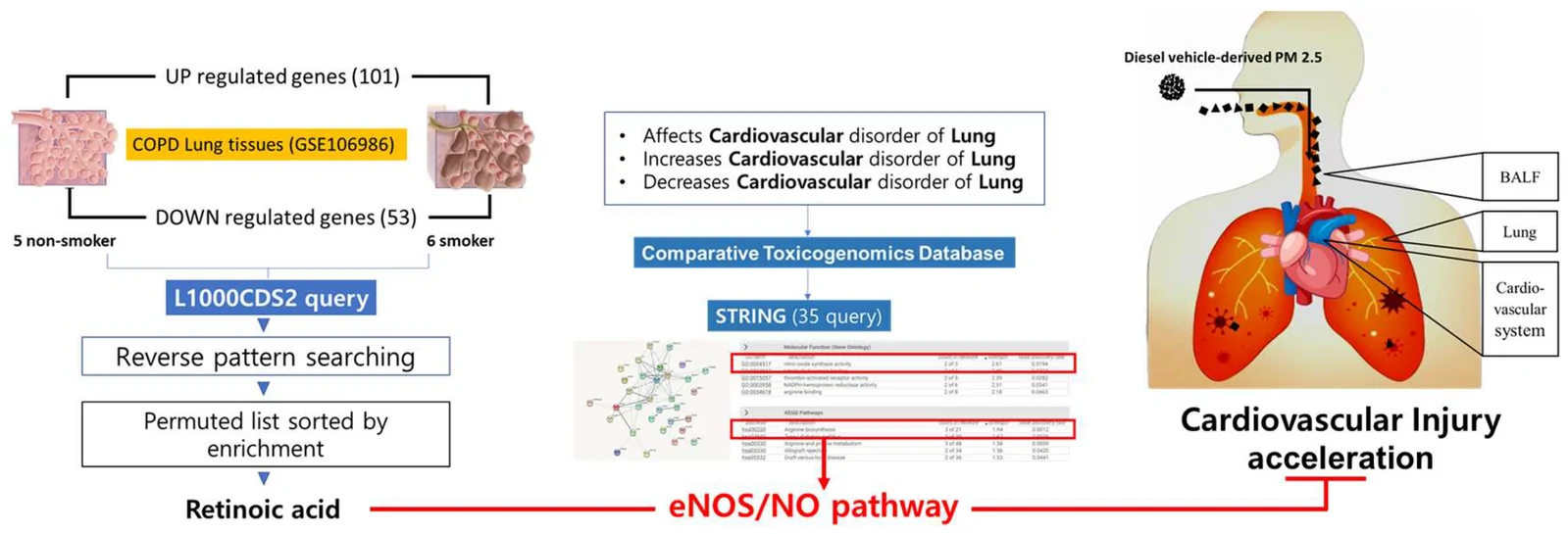
Identification of retinoid acid (RA) for the treatment of PM-induced acceleration of cardiovascular injury using in silico analysis. Differentially expressed genes edited from GSE106986 were applied to L1000CDS^2^ to obtain natural candidate agents with the potential to reverse pulmonary disorders. From the top 14-ranked chemicals, natural compound RA was selected. The comparative toxicogenomics database and string were used to search possible pathways for decreasing lung-related cardiovascular disorders. eNOS/NO pathway was possibly selected to treatment of PM-induced acceleration of cardiovascular injury induced by RA.

To identify genes potentially involved in the PM-induced acceleration of atherosclerosis, the GSE106986 dataset was obtained from the GEO database. The dataset comprised lung tissue samples from patients with chronic obstructive pulmonary disease (COPD) and non-smoking controls and was originally analyzed to identify differences in gene expression associated with COPD. The dataset included five non-smoking controls and six smokers with COPD (Figure 1). The gene symbols from the upregulated and downregulated gene sets were converted to the corresponding probe IDs based on the Agilent-026652 Whole Human Genome Microarray 4x44K v2 (Agilent Technologies, Santa Clara, CA, USA). The resulting DEG signatures were subsequently submitted to the L1000 Characteristic Direction Signature Search Engine (L1000CDS^2^) to identify candidate therapeutic compounds capable of reversing the gene expression profile associated with pulmonary injury.

### 2.2. Identification of Candidate Pathways Linking Pulmonary and Cardiovascular Disease (CVD)

To identify potential signaling pathways linking pulmonary injury to cardiovascular disorders, The CTD was used to investigate curated gene–disease associations between pulmonary and cardiovascular disorders. Three CTD queries were performed using the terms “affect cardiovascular disorder of lung”, “increase cardiovascular disorder of lung”, and “decreases cardiovascular disorder of lung”. A total of 35 associated genes were identified from these queries and subsequently subjected to molecular function and Kyoto Encyclopedia of Genes and Genomes (KEGG) pathway enrichment analyses using the STRING database. Based on the resulting gene–pathway associations, the endothelial nitric oxide synthase (eNOS)/nitric oxide (NO) signaling pathway was identified as a potential pathway involved in PM-induced acceleration of atherosclerosis and was subsequently selected for experimental validation of the protective effects of RA.

### 2.3. DEP and PM2.5 Sampling

DEPs used in the in vivo experiments were obtained from the Korea Automotive Technology Institute (KATECH, Cheonan, Republic of Korea), as described previously [16]. For the in vitro experiments, diesel particulate matter (PM2.5; SRM 2975) was purchased from the National Institute of Standards and Technology (NIST, Gaithersburg, MD, USA). DEPs were suspended in saline at 1 mg/mL, subjected to ultrasonication for 1 h, and subsequently allowed to stand for 24 h. Before use, the PM2.5 suspension was passed through a 2.0 μm syringe filter (SLAP05010, Millipore, Burlington, MA, USA).

### 2.4. Experimental Animals

Six-week-old male C57/BL6 mice and apolipoprotein-E gene-deficient mice (ApoE−/−) were obtained from Saeronbio. Inc. (Uiwang, Korea). The animals were subsequently maintained in cages under controlled environmental conditions, with the ambient temperature set at 22 ± 2 °C, relative humidity maintained at 50–60%, and a 12 h light/dark cycle. For dynamic inhalation exposure, the animals were transferred to a dynamic inhalation chamber equipped with an aerosol delivery system (Buxco^®^ Mass Dosing Controller, DSI, 461287, St. Paul, MN, USA). After a week of adaptation, the mice were randomly divided into five groups (*n* = 15 in each group): group I (control WT C57/BL6), group II (ApoE−/− saline only), group III (ApoE−/− DEP), group IV (ApoE−/− DEP + tretinoin 1 mg/kg/day), group V (ApoE−/− DEP + tretinoin 5 mg/kg/day). The animals were randomly assigned to the experimental groups. Both group allocation and outcome assessment were conducted under blinded conditions. All-trans retinoic acid (tretinoin) was orally administered for 8 weeks and exposed to DEP aerosols for 1 h per day in three exposure sessions excluding weekends. A total of 75 mice were randomly assigned to five experimental groups (*n* = 15 per group). Different subsets of animals were used for the various experimental analyses, depending on the type of outcome assessed. For each specific analysis, data were obtained from three animals per group (*n* = 3). No animals were excluded or lost during the study. Systolic blood pressure (SBP) was assessed noninvasively using the tail-cuff plethysmography technique, with measurements obtained via an automated sphygmomanometer (MK2000; Muromachi Kikai, Tokyo, Japan). All animal experiments were performed in compliance with the ethical standards established by the Wonkwang University Animal Experiment Ethics Committee (Confirmation No. WKU25-42). All animal procedures were conducted in accordance with the institutional guidelines and standard laboratory practices approved by the Wonkwang university. Appropriate measures were taken to minimize animal pain, suffering, and distress, and the animals were monitored in accordance with institutional requirements.

### 2.5. Plasma Biochemical Analysis

Mice were anesthetized with isoflurane. Blood was collected by cardiac puncture. Cervical dislocation was then performed to confirm death. Blood samples were collected in EDTA-containing vacutainer tubes and centrifuged at 3000 rpm for 20 min at 4 °C. The serum was then separated from the centrifuged samples. C-reactive protein (CRP) concentrations were determined using a sandwich enzyme-linked immunosorbent assay (ELISA) kit (PTX1; ab157712; Abcam, Cambridge, MA, USA), following the manufacturer’s protocol. The kit had a sensitivity of 0.159 μg/mL and a measurement range of 0.78–100 μg/mL. Total cholesterol (T-cho), low-density lipoprotein cholesterol (LDL-cho), very-low-density lipoprotein cholesterol (VLDL-cho), and high-density lipoprotein cholesterol (HDL-cho) were quantified using commercially available assay kits from Abcam. Triglyceride (TG) concentrations were measured using a commercial assay kit (AM157S-K; Asanpharm, Yeongcheon, Republic of Korea). The atherogenic index was calculated as follows: (Total cholesterol − HDL cholesterol)/HDL cholesterol.

### 2.6. Bronchoalveolar Lavage Fluid Collection and Analysis

Following 48 h of DEP exposure, 0.5 mL of PBS was instilled into the lungs to collect bronchoalveolar lavage fluid (BALF). The collected BALF was centrifuged at 400× *g* for 10 min at 4 °C, and the resulting supernatant was stored at −60 °C until cytokine analysis. CXCL1 (MKC00B) and CXCL2 (MM200) concentrations were determined using ELISA kits from R&D Systems (Quantikine; Minneapolis, MN, USA). Angiogenic and angiostatic chemokines were quantified in accordance with the manufacturer’s instructions.

### 2.7. Quantitative Polymerase Chain Reaction (qPCR)

RNA was extracted from tissue samples using the RiboEx™ reagent kit (GeneAll Biotechnology, Seoul, Republic of Korea). The extracted RNA served as the template for cDNA synthesis, which was performed with the HelixCript™ Easy cDNA Synthesis kit (NanoHelix, Daejeon, Republic of Korea). Quantitative real-time PCR (qPCR) was subsequently conducted using a StepOnePlus Real-Time PCR System (Applied Biosystems, Foster City, CA, USA) in combination with the RealHelix Premier qPCR kit (NanoHelix, Daejeon, Republic of Korea). The procedures for RNA extraction, reverse transcription, and qPCR analysis followed the respective manufacturers’ instructions.

### 2.8. Histological Analysis

Mice were anesthetized with isoflurane. Lung and aorta tissues were subsequently harvested. Cervical dislocation was then performed to confirm death. Upon completion of the experiment, the lung and carotid artery specimens were harvested, immersed in 10% neutral buffered formalin (NBF) for fixation, and subsequently processed for paraffin embedding. Paraffin-embedded samples were then serially sectioned at 4 µm. The tissue sections were stained with hematoxylin and eosin (H and E) and Masson’s trichrome. Micrographs (EasyScan Pro 6 digital slide scanner, Motic, Xiamen, China) were taken and arterial wall thickness was evaluated from three randomly selected sections per sample. Images were acquired at magnifications of 100× or 200×.

### 2.9. TEM Observation of Vascular Lesions

Transmission electron microscopy (TEM) was used to examine the ultrastructural features of the lesions with a JEM-2100 microscope (JEOL, Tokyo, Japan). The first branch of the aorta was initially immersed in 2.5% glutaraldehyde overnight for fixation, followed by washing with 0.1 M phosphate buffer. The tissue was then postfixed with osmium tetroxide for 2 h, dehydrated through a graded ethanol series and acetone, and embedded in epoxy resin. Ultrathin sections measuring 50 nm in thickness were subsequently prepared and examined by TEM.

### 2.10. Western Blot Analysis

Tissue samples were homogenized in radioimmunoprecipitation assay (RIPA) buffer supplemented with a Halt™ protease inhibitor cocktail (Thermo Fisher Scientific, Cleveland, OH, USA) to obtain total protein extracts. The buffer was obtained from ELPIS-Biotech (Daejeon, Republic of Korea). Protein concentrations were quantified using the Bradford method. Equal amounts of protein (30 µg per sample) were resolved on 10% SDS–polyacrylamide gels and subsequently transferred onto polyvinylidene fluoride (PVDF) membranes. Membrane blocking was performed with 5% (*w*/*v*) skim milk for 1 h at room temperature. Following the washing step, the membranes were incubated with the primary antibody overnight at 4 °C and then exposed to the secondary antibody for 1 h at room temperature. Primary antibodies were used at the following concentrations: ICAM-1 (#4915, 1:1000), VCAM-1 (#14694, 1:1000), p-Akt (#9271, 1:1000), Akt (#9272, 1:1000), p-eNOS (#9571, 1:1000), eNOS (#32027, 1:1000), GTPCH-1 (#36788, 1:1000), and β-actin (1:1000). Primary antibodies, except where otherwise specified, were purchased from Cell Signaling Technology, Inc. (Danvers, MA, USA). Immunoreactive protein bands were visualized using an enhanced chemiluminescence (ECL) reagent (Kindle Biosciences, Greenwich, CT, USA), and the resulting signals were captured with a ChemiDoc imaging system (Bio-Rad, Hercules, CA, USA).

### 2.11. Cell Cultures

Human umbilical vein endothelial cells (HUVECs) were sourced from PromoCell (Heidelberg, Germany) and maintained in Endothelial Cell Basal Medium 2 from the same supplier at a density of 5 × 10^5^ cells/mL. Cell cultures were kept at 37 °C in a humidified incubator with an atmosphere of 5% CO_2_ and 95% air. Cells were treated with PM (100 µg/mL) and TNF-alpha (50 ng/mL) for 24 h with/without RA (1, 2, and 5 µm, respectively).

### 2.12. BH2 and BH4 Measurements

Frozen aortic tissue specimens were analyzed to determine total tetrahydrobiopterin (BH4) and dihydrobiopterin (BH2) levels. The tissue samples were homogenized in 5% trichloroacetic acid (TCA), followed by centrifugation at 15,000× *g* for 15 min at room temperature. The resulting supernatants were collected for subsequent analysis. BH2 and BH4 concentrations were quantified using commercially available assay kits (Novus Biologicals, Centennial, CO, USA; NBP2-74982 and NBP2-74983). Absorbance was recorded at 405 nm using a microplate reader (Tecan, Männedorf, Switzerland) and the results were expressed as pg/mL of protein.

### 2.13. Statistical Analysis

Quantitative results are reported as mean ± standard deviation (SD), based on at least three independent experiments. Differences among experimental groups were evaluated using one-way analysis of variance (ANOVA), with Tukey’s post hoc multiple-comparison procedure applied for pairwise comparisons. Statistical analyses were conducted in GraphPad Prism (version 8.0; GraphPad Software, San Diego, CA, USA).

## 3. Results

### 3.1. Identification of Retinoic Acid as a Candidate Therapeutic Compound and Its Potential Mechanism by Bioinformatic Analysis

L1000CDS^2^ was used to identify potential therapeutic compounds by comparing the DEG signature with reference gene expression signatures in the L1000 database and ranking chemical perturbations according to their ability to reverse the disease-associated gene expression pattern. To obtain the disease-associated gene expression signature, we utilized lung tissue gene expression data from the GEO dataset GSE106986, which included five non-smokers and six smokers (Supplementary Materials S1). A total of 154 DEGs were identified based on the predefined thresholds of logFC > 1.15 for upregulated genes and logFC < −1.5 for downregulated genes, including 101 upregulated and 53 downregulated genes (Table S1). The resulting DEG signature was analyzed using L1000CDS^2^, and 14 top-ranked chemical perturbation signatures were identified (Table S2, Supplementary Materials S2). Because our aim was to identify a natural compound with potential protective effects against PM-induced cardiovascular injury, synthetic chemical compounds were excluded from further consideration. Finally, RA, a biologically active metabolite of vitamin A, was selected as a potential therapeutic candidate for the PM-induced acceleration of atherosclerosis (Figure 1).

### 3.2. Identification of the Nitric Oxide-Related Signaling as a Candidate Pathway Linking Pulmonary and CVD

To identify potential molecular pathways linking pulmonary injury to CVD, the 35 genes obtained from the CTD analysis were subjected to protein–protein interaction (PPI) and functional enrichment analyses using the STRING database (Table S3). Among the 35 genes, 32 were mapped to the STRING interaction network, which showed significantly greater protein–protein interactions than expected by chance (PPI enrichment *p* < 1.0 × 10^−16^) (Supplementary Materials S3). Functional enrichment analysis identified several biological processes and molecular functions associated with inflammation and cardiovascular regulation (Supplementary Materials S4). Notably, nitric oxide synthase activity was significantly enriched in the molecular function category (two of three genes; strength = 2.61; FDR = 0.0194). In addition, KEGG pathway analysis also identified several significantly enriched pathways, including arginine biosynthesis (3 of 21 genes; strength = 1.94; FDR = 0.0012) and arginine and proline metabolism (3 of 48 genes; strength = 1.58; FDR = 0.0059). These findings indicated that nitric oxide-related molecular functions and pathways were significantly associated with the genes linking pulmonary and CVD. Based on these bioinformatic findings, the endothelial nitric oxide synthase (eNOS)/nitric oxide (NO) signaling pathway was selected as a candidate mechanism for subsequent experimental validation of the protective effects of RA against PM-induced acceleration of atherosclerosis.

### 3.3. Effect of RA on Blood Pressure and Lipid Measurements

**Figure 2:**
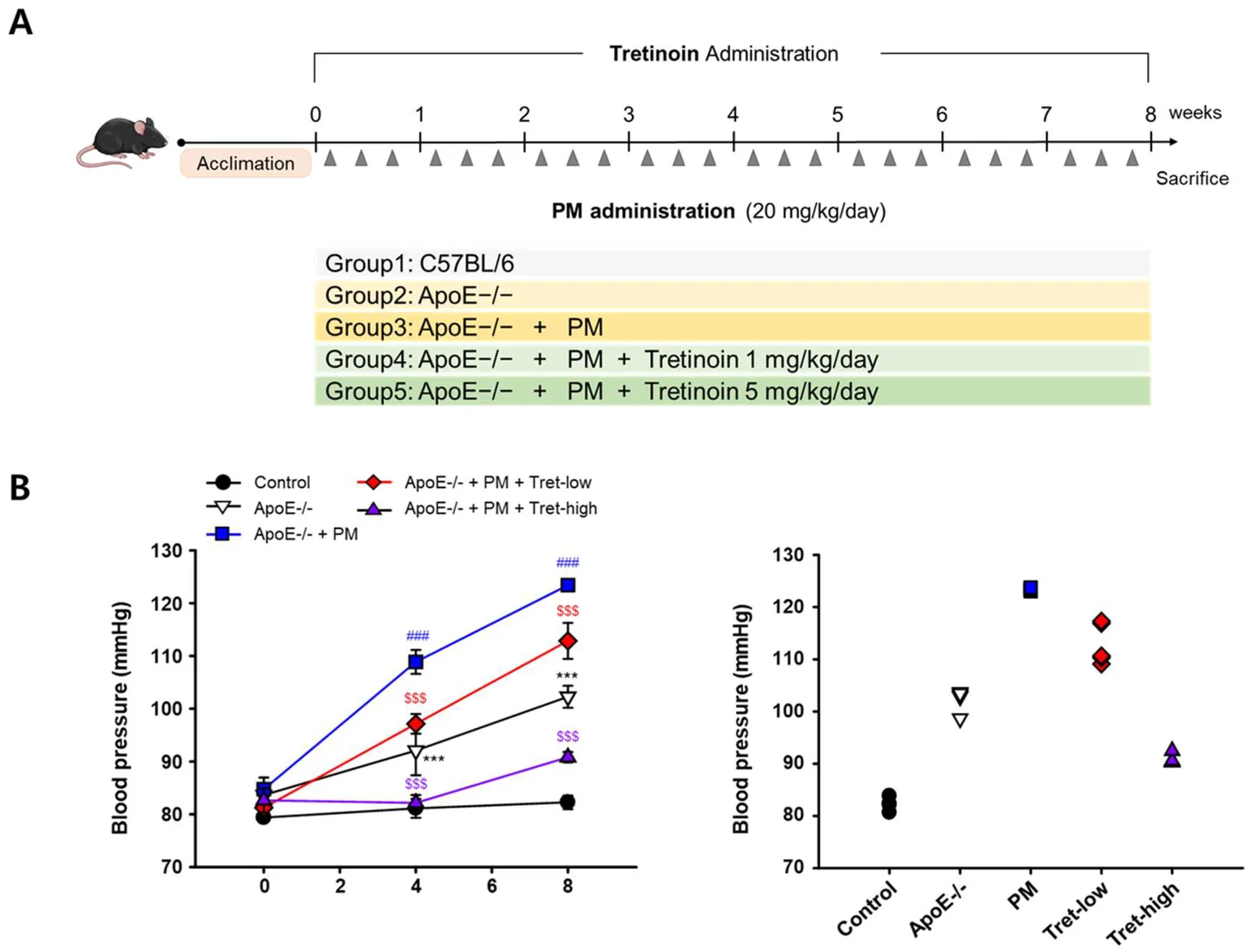
Ameliorative effect of RA on PM-induced changes in blood pressure. (**A**) Schematic diagram of accelerated cardiovascular injury in diesel exhaust-derived PM2.5-exposed ApoE−/− mice. Male six-week-old mice were randomly divided into five groups (*n* = 15 in each group): group I (control WT C57/BL6), group II (ApoE−/− saline only), group III (ApoE−/− DEP), group IV (ApoE−/− DEP + tretinoin 1 mg/kg/day), group V (ApoE−/− DEP + tretinoin 5 mg/kg/day). All-trans retinoic acid (tretinoin) was orally administered for 8 weeks. Mice were exposed to DEP aerosols for 1 h per day in three exposure sessions using a Buxco^®^ Mass Dosing Controller. (**B**) Blood pressure by tail cuff measurements in control WT and ApoE−/− mice. Data are presented as means ± SD. *** *p* < 0.001 vs. control; ### *p* < 0.001 vs. ApoE−/−; $$$ *p* < 0.001 vs. ApoE−/− PM.

The levels of systolic blood pressure from all experimental groups were approximately 80–85 mmHg at the starting point of study. After 8 weeks, systolic blood pressure in the PM-inhaled ApoE−/− was further increased compared with ApoE−/− (*p* < 0.01). However, in the RA group, blood pressure was significantly decreased compared to the PM-inhaled ApoE−/− group (Figure 2A). As shown in Table 1, PM-treated ApoE−/− group significantly increased urine volume and serum creatinine and were decreased by administration of RA. Food, water intake, and body weights were not significantly different. PM-exposed ApoE−/− mice.

PM-inhaled ApoE−/− group showed increased total cholesterol, triglyceride, LDL cholesterol, and hs-CRP levels. However, these levels were significantly decreased by treatment with RA. RA also decreased the atherogenic index in PM-inhaled ApoE−/− mice. The plasma levels of HDL-cholesterol levels by RA administration significantly increased compared to PM-inhaled ApoE−/− mice (Table 2).

### 3.4. Effect of RA on CXCL1 and CXCL2 Levels in Bronchoalveolar Lavage Fluid

PM inhalation further increases BAL leukocyte count levels in ApoE−/− mice. The expression levels of CXCL1 and CXCL2 were significantly increased by PM exposure. The number of leukocytes and the proportion of neutrophils in PM-inhaled ApoE−/− mice were significantly higher than control or ApoE−/− mice. However, the levels of CXCL1 and CXCL2 in BALF were effectively downregulated in RA treated groups, suggesting that RA has a protective effect on epithelial barrier function in BALF against PM inhalation.

### 3.5. Effect of RA on PM-Induced Pulmonary Injury

**Figure 3:**
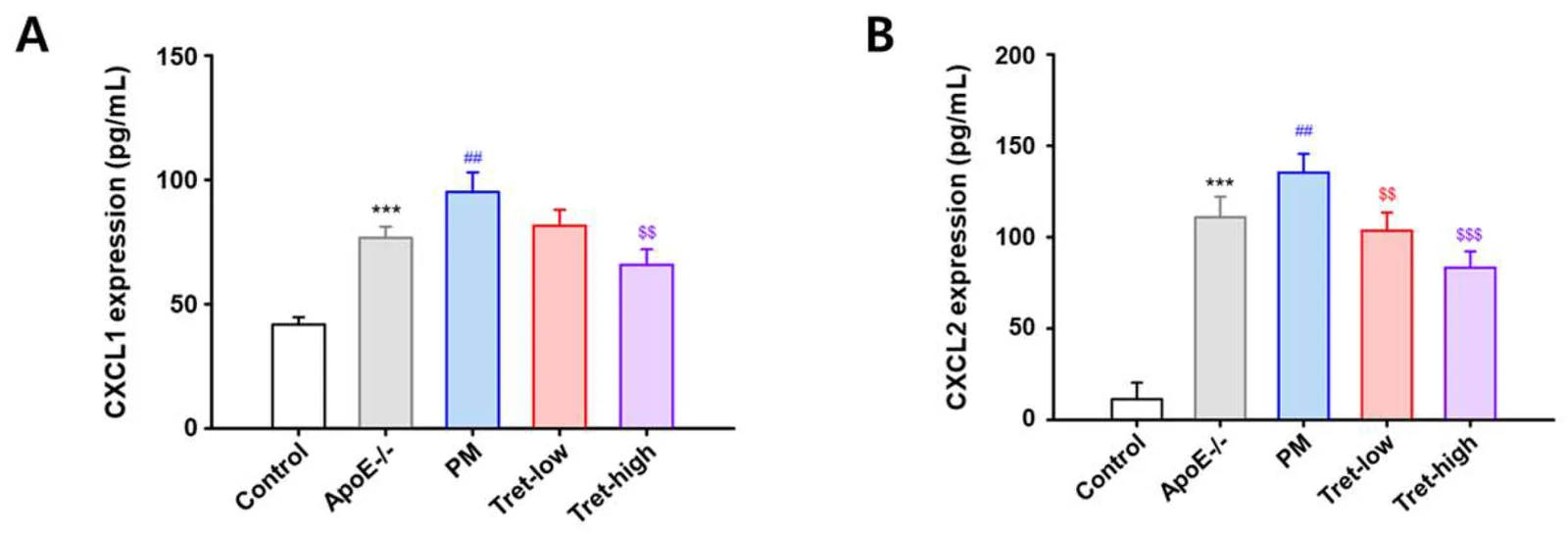
Effects of RA on PM-induced CXCL1 (**A**) and CXCL2 (**B**) production in bronchoalveolar lavage fluid (BALF). Neutrophils were studied by determining the levels of the CXCL1, CXCL2 with the ELISA. Data are presented as means ± SD. *** *p* < 0.001 vs. control; ## *p* < 0.01 vs. ApoE−/−; $$ *p* < 0.01, $$$ *p* < 0.001 vs. ApoE−/− PM.

**Figure 4:**
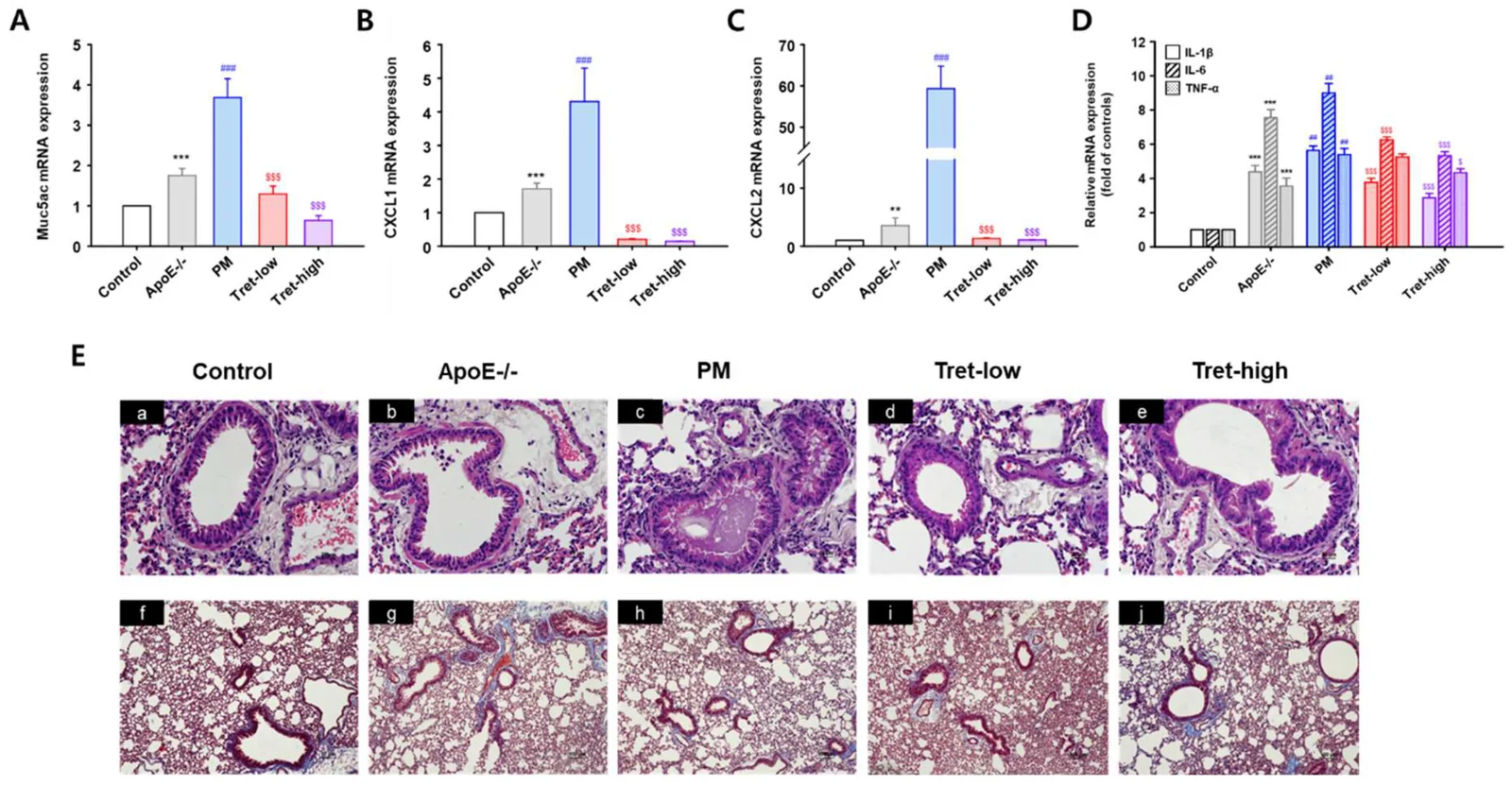
Effect of RA on PM-induced lung injury. Muc5ac (**A**), CXCL1 (**B**), CXCL2 (**C**) mRNA expression level was measured. (**D**) inflammatory factors mRNA expression of IL-1β, IL-6, TNF-α. (**E**) Measurements of H and E (upper) and Masson’s trichrome (lower) staining of lung tissue from the indicated groups of mice (a–e, scale bars = 20 µm, f–j, 100 µm). Data are presented as means ± SD. ** *p* < 0.05, *** *p* < 0.001 vs. control; ## *p* < 0.01, ### *p* < 0.001 vs. ApoE−/−; $ *p* < 0.05, $$$ *p* < 0.001 vs. ApoE−/− PM.

To investigate whether RA reverse pulmonary inflammation, cell adhesion molecules and cytokine expression were examined by ELISA or real-time PCR. In the BALF, the increase in CXCL1 and CXCL2 in PM-exposed ApoE−/− mice was reduced by RA (Figure 3). As shown in Figure 4A–C, RA significantly reduced the mRNA levels of Muc5ac, CXCL1, and CXCL2 of lung tissues. IL-1β, IL-6, and TNF-α mRNA levels also were upregulated in exposure to PM; however, RA reversed those expression levels (Figure 4D).

Histopathological changes in lung were evaluated by H and E and Masson’s trichrome staining to determine the effects of DEP exposure and RA treatment on pulmonary inflammation. PM-exposed ApoE−/− mice exhibited apparent pulmonary inflammatory changes, characterized by increased inflammatory cell infiltration around the bronchiolar and perivascular regions, thickening of the alveolar septa, and disruption of the normal alveolar architecture. These histopathological changes were markedly attenuated in mice treated with RA at both 1 and 5 mg/kg/day, with reduced inflammatory cell infiltration and improved preservation of the bronchiolar and alveolar structures (Figure 4E).

### 3.6. Effect of RA on the Vascular Inflammation

**Figure 5:**
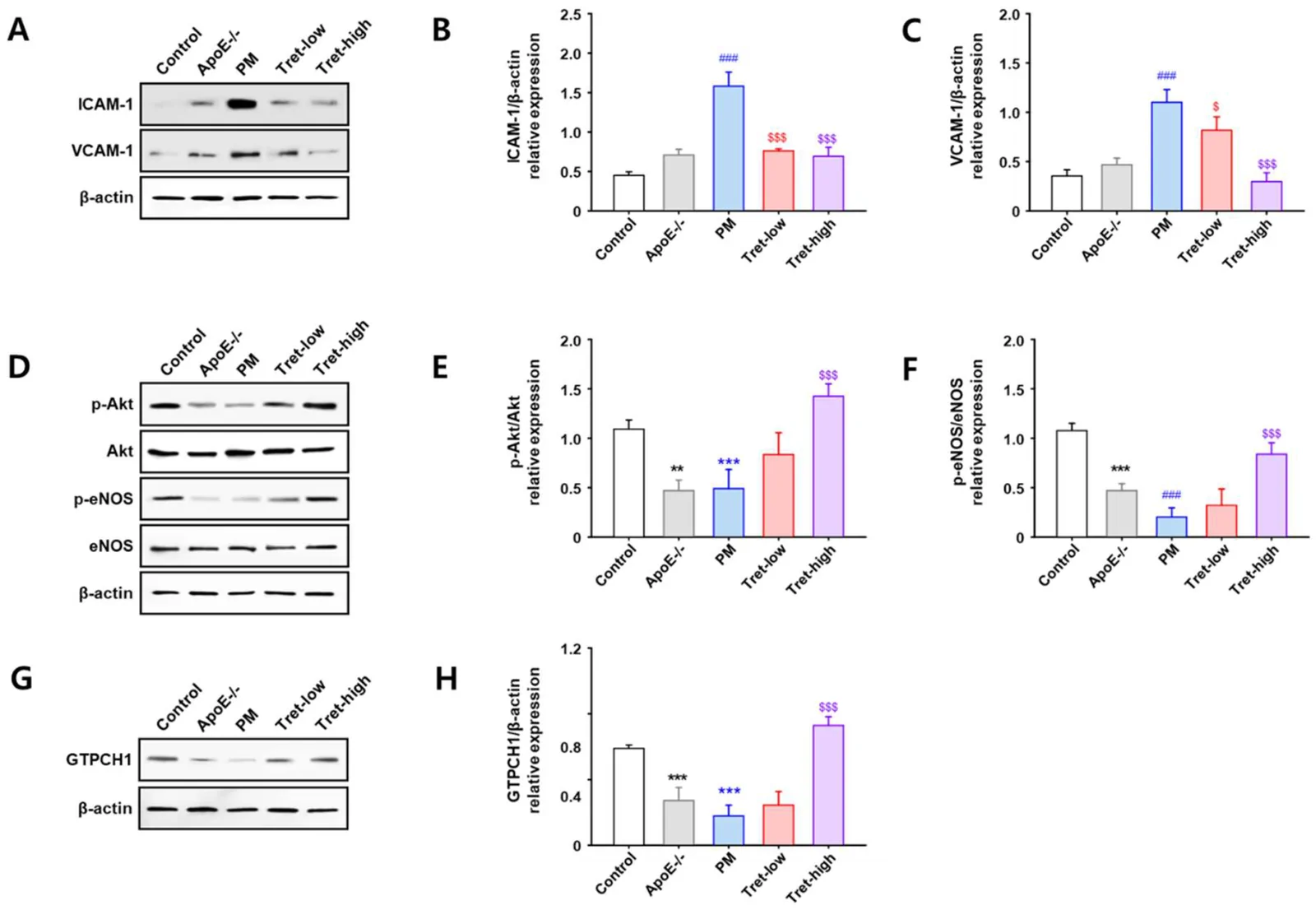
Effects of RA on cell adhesion molecules, eNOS, and GTPCH1 expression in the aortas of ApoE KO mice. (**A**) Protein levels of the ICAM-1, VCAM-1 were determined by Western blot analysis. The graphs represent the ratios of ICAM-1 (**B**) and VCAM-1 (**C**) protein levels to β-actin protein levels. (**D**) Representative Western blot images showing both total and phosphorylated forms of Akt and eNOS. Graphs represent the ratios of phosphorylated to total protein levels of (**E**) Akt and (**F**) eNOS. (**G**) Protein levels of the GTPCH1 were determined by Western blot analysis. The graphs represent the ratios of GTPCH1 protein levels to β-actin protein levels (**H**). Data are presented as means ± SD. ** *p* < 0.05, *** *p* < 0.001 vs. control; ### *p* < 0.001 vs. ApoE−/−; $ *p* < 0.05, $$$ *p* < 0.001 vs. ApoE−/− PM.

Western blot analysis was performed to evaluate the effects of RA on endothelial activation and the Akt/eNOS signaling pathway in thoracic aortic tissues. DEP exposure markedly increased the expression of the endothelial adhesion molecules ICAM-1 and VCAM-1, whereas RA treatment at 1 or 5 mg/kg/day attenuated these increases. DEP exposure also altered Akt and eNOS phosphorylation, while RA treatment restored p-Akt and p-eNOS levels. In addition, RA treatment markedly increased the expression of GTP cyclohydrolase-1 (GTPCH-1), a key enzyme involved in BH4 biosynthesis and eNOS coupling. These findings indicate that RA suppresses DEP-induced endothelial activation and improves vascular endothelial function through modulation of the GTPCH-1/Akt/eNOS signaling pathway (Figure 5).

### 3.7. Effect of RA on PM-Induced Vascular Structural Damage

**Figure 6:**
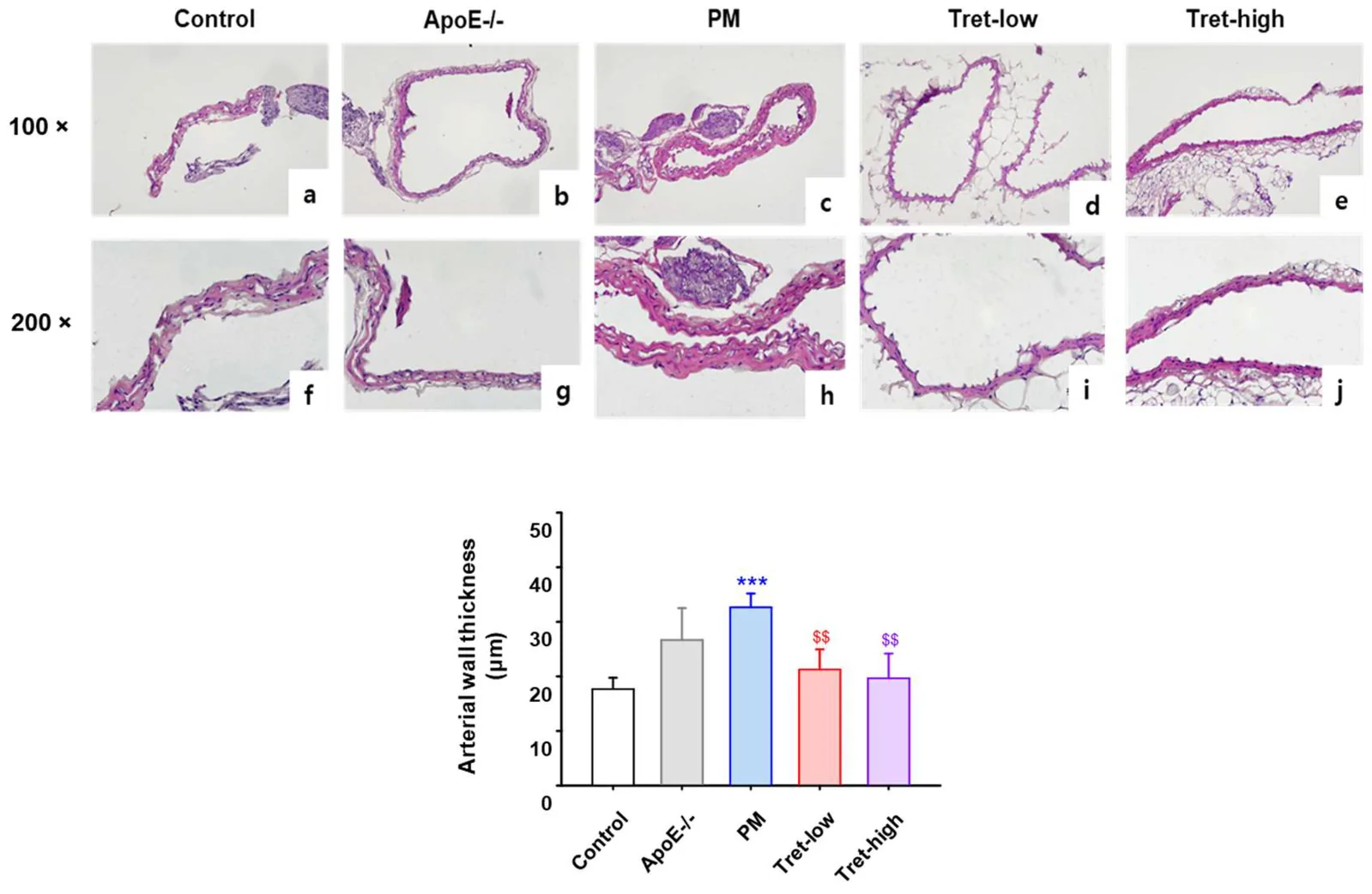
Effects of RA on histopathological alterations in blood vessel of diesel exhaust-derived PM2.5-exposed ApoE−/− mice. Following H and E staining, arterial wall thickness was measured (lower panel). Histological analysis of carotid artery using H and E staining (magnification (**a**–**e**), 100×, (**f**–**j**), 200×). Data are presented as means ± SD. *** *p* < 0.001 vs. control; $$ *p* < 0.005 vs. ApoE−/− PM.

Histopathological analysis of the carotid artery revealed marked increased aortic wall thickness and disruption of the normal vascular structure in PM-exposed ApoE−/− mice compared with control or ApoE−/− group. Figure 6 showed that treatment with RA at 1 or 5 mg/kg/day attenuated DEP-induced aortic wall thickening. Foam cells, migrated smooth muscle cells, and a large quantity of cholesterol crystals and necrotic substances were also observed in the lesions. Quantitative histomorphometric analysis further confirmed the inhibitory effect of RA on DEP-induced vascular wall thickening. These findings suggest that RA protects against DEP-induced structural alterations in the aorta.

**Figure 7:**
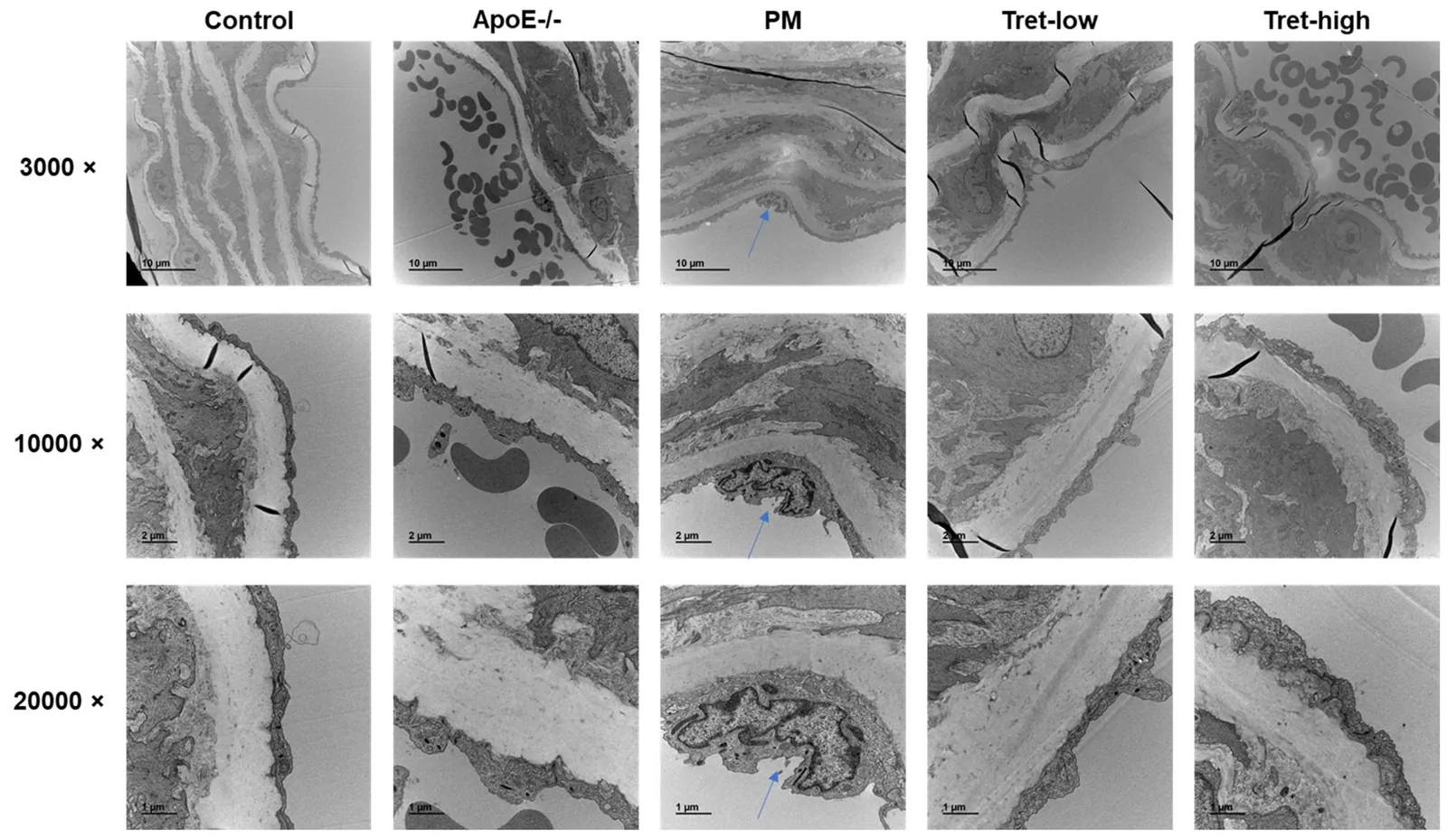
Effect of RA on histological morphology changes. Representative TEM picture of the plaque in carotid artery. Foam cells, migrated smooth muscle cells, and a large quantity of cholesterol crystals and necrotic substances (blue arrow) were observed in the lesions of mice in diesel exhaust-derived PM2.5-exposed ApoE−/− mice. Magnification showed 3000×, 10,000×, 20,000× respectively. (Scale bar = 10, 2, 1 μm).

TEM was performed to evaluate ultrastructural changes in the carotid artery. Compared with ApoE−/− mice, PM-exposed ApoE−/− mice showed more pronounced endothelial damage, characterized by disruption and irregularity of the endothelial lining and alterations in the underlying vascular wall architecture. These ultrastructural abnormalities were markedly attenuated by treatment with RA at 1 or 5 mg/kg/day, with the higher dose showing a more evident preservation of endothelial integrity and vascular wall structure. These findings indicate that PM exposure exacerbates vascular ultrastructural damage in ApoE−/− mice, whereas RA alleviates DEP-induced vascular injury (Figure 7).

### 3.8. RA Protects Against PM-Induced Endothelial Dysfunction by Restoring eNOS Coupling

**Figure 8:**
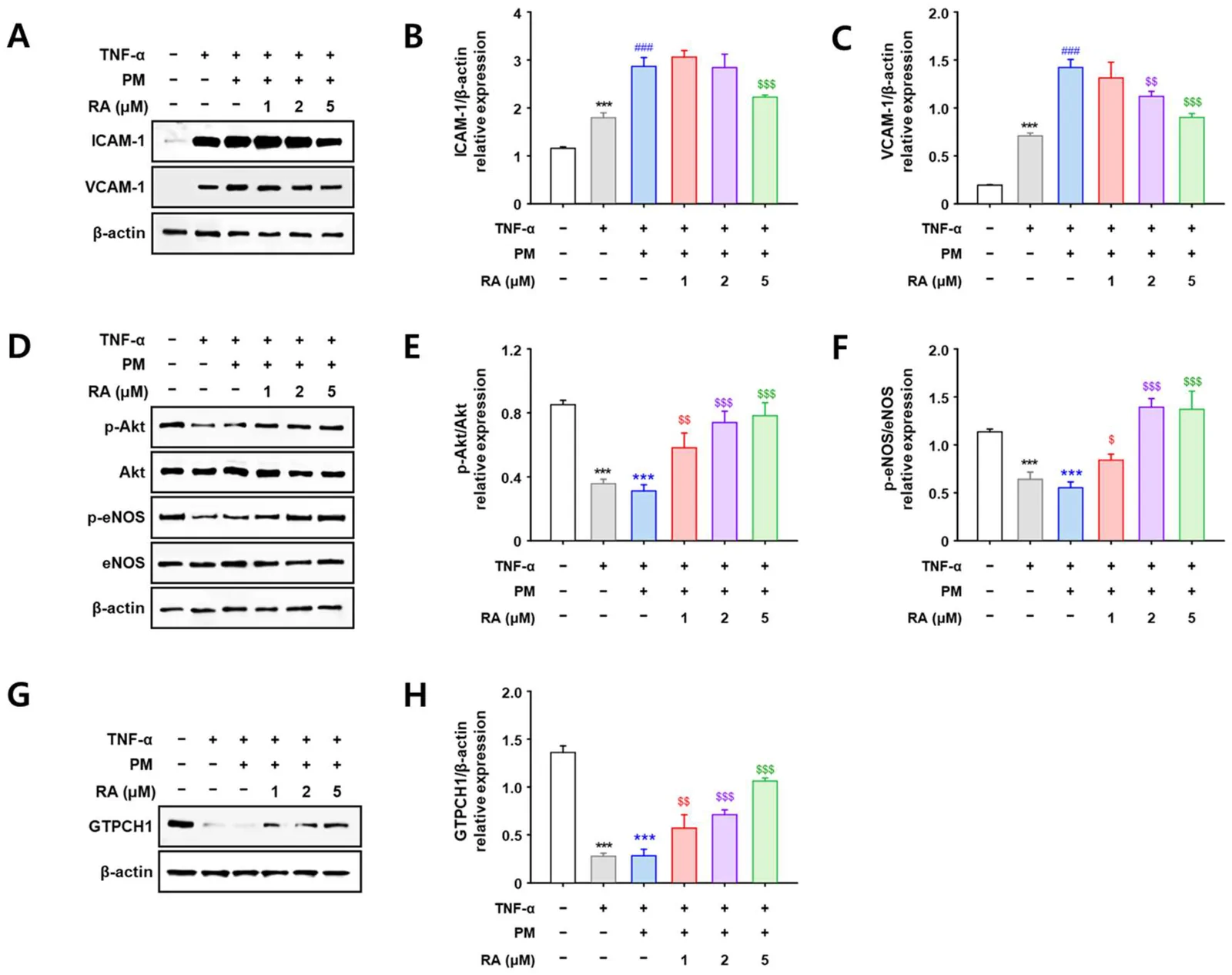
Effects of RA on cell adhesion molecules, eNOS, and GTPCH1 expression in PM-exposed HUVECs. (**A**) Protein levels of the ICAM-1, VCAM-1 were determined by Western blot analysis. The graphs represent the ratios of ICAM-1 (**B**) and VCAM-1 (**C**) protein levels to β-actin protein levels. (**D**) Representative Western blot images showing both total and phosphorylated forms of Akt and eNOS. Graphs represent the ratios of phosphorylated to total protein levels of (**E**) Akt and (**F**) eNOS. (**G**) Protein levels of the GTPCH1 were determined by Western blot analysis. The graphs represent the ratios of GTPCH1 protein levels to β-actin protein levels (**H**). Data are presented as means ± SD (*n* = 3). *** *p* < 0.001 vs. control; ### *p* < 0.001 vs. ApoE−/−; $ *p* < 0.05, $$ *p* < 0.01, $$$ *p* < 0.001 vs. ApoE−/− PM.

To further confirm the vascular protective effects of RA at the cellular level, HUVECs were examined for changes in endothelial inflammatory markers and signaling pathways. PM exposure markedly increased the expression of ICAM-1 and VCAM-1, whereas RA treatment attenuated these increases. In addition, RA restored PM-induced alterations in Akt and eNOS phosphorylation and increased GTPCH-1 expression (Figure 8).

**Figure 9:**
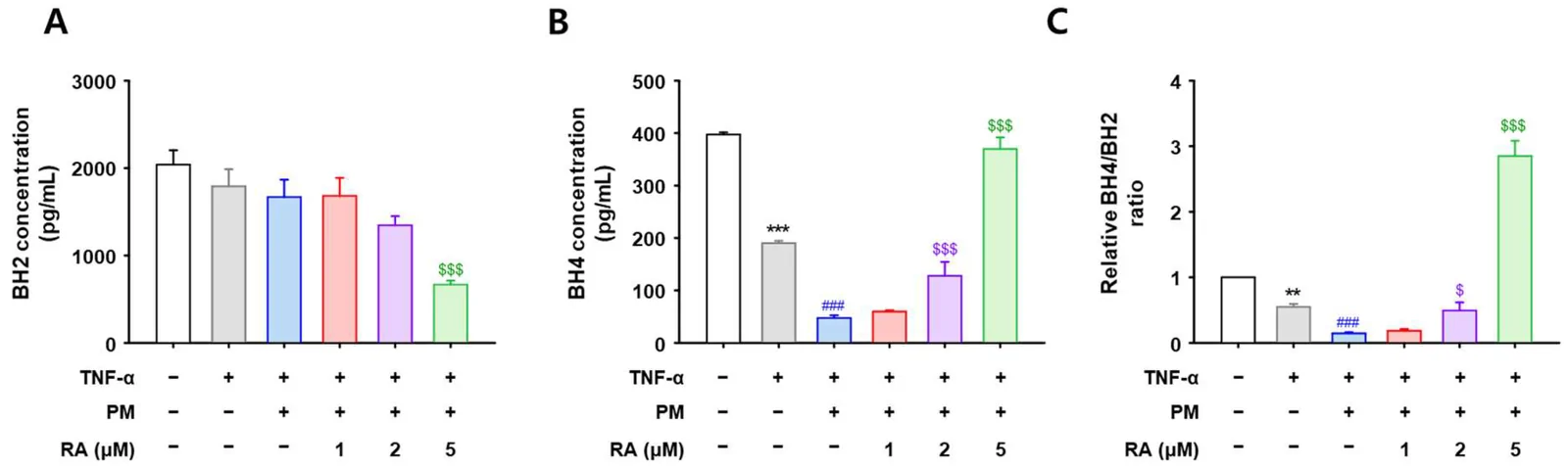
Effect of RA on eNOS coupling in PM-exposed HUVECs. HUVECs were pretreated with RA (1–5 μM), and TNF-α (50 ng/mL) with/without PM (100 μg/mL). (**A**) Quantification of BH2 determined using a dihydrobiopterin ELISA kit. (**B**) Quantification of BH4 determined using a tetrahydrobiopterin ELISA kit. (**C**) Ratio of BH4 to BH2 in HUVECs. Data are presented as means ± SD (*n* = 3). ** *p* < 0.01, *** *p* < 0.001 vs. control; ### *p* < 0.001 vs. ApoE−/−; $ *p* <0.05, $$$ *p* < 0.001 vs. ApoE−/− PM.

We next examined intracellular BH2 and BH4 levels to determine whether RA-mediated GTPCH-1/eNOS expression was associated with alterations in biopterin homeostasis. PM markedly decreased BH4 levels and the BH4/BH2 ratio, whereas RA treatment restored BH4 levels and significantly increased the BH4/BH2 ratio. In contrast, RA reduced BH2 levels, indicating a shift toward a more favorable biopterin redox balance. These results suggest that RA preserves BH4 availability and maintains the BH4/BH2 balance, thereby promoting eNOS coupling under PM-induced endothelial vascular injury (Figure 9).

## 4. Discussion

Air pollution such as PM2.5 is increasingly recognized as an important risk factor for CVD [17,18]. It is necessary to search for natural resources for the successful treatment of the PM-induced acceleration of atherosclerosis. In this study, we used a bioinformatics approach to identify a candidate agent to reverse the progression of COPD in humans. RA, a biologically active metabolite, was selected as a possible therapeutic candidate, and we validated its potential as a therapeutic agent for vascular dysfunction leading to PM-induced acceleration of atherosclerosis. We demonstrated that DEP exposure aggravated pulmonary inflammation and cardiovascular abnormalities in ApoE−/− mice, whereas RA treatment attenuated these pathological changes. These findings indicate that PM exposure can exacerbate cardiovascular injury and RA may provide protective effects against PM-induced injury.

PM is also similar risk factors to atherosclerosis similar to age, hypertension, and hypercholesterolemia [19,20]. DEP exposure adversely affected systemic cardiovascular and metabolic parameters in ApoE−/− mice, as evidenced by increased systolic blood pressure and atherogenic lipid profiles. RA treatment improved these abnormalities. All-trans retinoic acid also attenuates the progression of Ang II-induced abdominal aortic aneurysms in ApoE−/− mice [11]. These findings suggest that RA attenuates the PM-induced acceleration of atherosclerosis by improving systemic cardiovascular and metabolic abnormalities.

The main biological targets of inhaled DEP are pulmonary epithelial cells and macrophages [21,22]. Respiratory exposure to DEP causes airway epithelial cell damage. DEP exposure induced a marked inflammatory response in the and BALF and lung, as evidenced by increased CXCL1, CXCL2, Muc5ac, IL-1β, IL-6, and TNF-α expression. RA treatment attenuated these pulmonary inflammatory responses. In vascular endothelial cells, DEP exposure increased the expression of the adhesion molecules ICAM-1 and VCAM-1, whereas RA treatment reduced their expression. Similar effects were observed in PM-exposed HUVECs, suggesting that RA suppresses PM-induced endothelial activation. Consistent with these anti-inflammatory effects, histopathological analysis revealed increased vascular wall thickness and disruption of the normal vascular structure in PM-exposed ApoE−/− mice compared with ApoE−/− mice, while RA treatment attenuated these alterations. TEM analysis further demonstrated more pronounced endothelial and vascular ultrastructural damage following PM exposure, which was also ameliorated by RA treatment. These findings suggest that the anti-inflammatory effects of RA may contribute to its protective effects against PM-induced vascular injury by preserving vascular structure and improving vascular inflammation.

eNOS/NO signaling as a potential link between pulmonary and cardiovascular disorders was selected by using CTD-based pathway analysis to identify the possible pathways as a potential link between pulmonary and cardiovascular disorders. Impaired eNOS signaling and altered BH4 homeostasis are involved in PM-induced endothelial dysfunction [23,24]. PM exposure reduced Akt and eNOS phosphorylation and decreased GTPCH-1 expression, accompanied by reduced BH4 levels and an unfavorable BH4/BH2 balance. Because BH4 is an essential cofactor for maintaining eNOS in its coupled state, the depletion or oxidation of BH4 may promote eNOS uncoupling and endothelial dysfunction. RA restored Akt/eNOS phosphorylation and GTPCH-1 expression and improved BH4 availability and the BH4/BH2 ratio. These findings suggest that RA may preserve endothelial function by maintaining GTPCH-1/BH4-dependent eNOS coupling under PM-induced vascular injury. Although our findings support the involvement of the GTPCH-1/BH4/eNOS coupling pathway, direct measurements of eNOS-derived NO or superoxide were not performed. Further studies using the pharmacological or genetic manipulation of this pathway will be necessary to confirm its contribution to the protective effects of RA. Thus, the present findings demonstrate that RA attenuates PM-induced pulmonary inflammation and the exacerbation of atherosclerotic vascular injury and suggest that the preservation of GTPCH-1/BH4-dependent eNOS coupling is an important mechanism underlying these protective effects.

The limitations of this study include the absence of RA-only treatment in ApoE−/− mice without DEP exposure and the lack of evaluation of DEP exposure and RA treatment in C57BL/6 mice. In future studies, we plan to investigate these conditions to further clarify the independent effects of RA and the effects of DEP exposure in mice without ApoE deficiency. Furthermore, the ApoE−/− mice were not fed a high-fat diet, and Oil Red O staining of the aortic arch, aortic root, or entire aorta was not performed. These limitations prevented a comprehensive assessment of atherosclerotic lesion burden and distribution. Future studies incorporating high-fat-diet feeding and Oil Red O staining are warranted to further clarify the effects of DEP exposure and RA treatment on atherosclerosis. Although mouse models are widely used in preclinical research, differences between mice and humans limit the direct generalization of these findings to human biology. Further studies, including clinical investigations, are needed to confirm the efficacy and safety of RA and its potential to protect against environmental exposures in humans.

## 5. Conclusions

Our findings demonstrate that RA attenuates PM-induced pulmonary inflammation and the exacerbation of atherosclerotic vascular injury in ApoE−/− mice. RA also improved systemic cardiovascular and metabolic abnormalities and reduced endothelial inflammatory activation. These findings suggest that the vascular protective effects of RA may be associated with modulation of the GTPCH-1/BH4/eNOS/NO signaling pathway, although further functional studies are needed to confirm the involvement of eNOS coupling. In conclusion, these findings confirmed RA as a potential therapeutic candidate for preventing the PM-induced acceleration of atherosclerosis and provide new insight into the role of eNOS coupling in PM-associated cardiovascular injury.

## Figures and Tables

**Table 1:** Effects of DEP exposure and RA treatment on physiological and renal function parameters in mice. Food intake, water intake, body weight, urine volume, blood urea nitrogen (BUN), creatinine, and albumin levels were measured in each experimental group. Data are presented as mean ± SEM. * *p* < 0.05, ** *p* < 0.01 vs. control.

	Control	ApoE−/−	PM	Tret-Low	Tret-High
Food intake (g)	35.92 ± 2.71	39.23 ± 2.01	32.74 ± 2.14	37.18 ± 0.74	39.22 ± 1.35
Water intake (g)	45.08 ± 1.57	42.92 ± 2.71	41.48 ± 1.87	43.23 ± 2.07	49.18 ± 0.74
Body weight (g)	25.72 ± 0.43	22.74 ± 0.47	22.05 ± 0.43	24.18 ± 0.41	25.11 ± 1.14
Urine volume (mL)	1.55 ± 0.57	2.36 ± 1.10	5.41 ± 2.11 *	4.62 ± 0.57 *	4.22 ± 0.58 *
BUN (mg/dL)	21.50 ± 1.31	24.03 ± 2.3	29.08 ± 1.5	18.45 ± 0.5	19.53 ± 0.86
Creatinine (mg/dL)	0.16 ± 0.04	0.37 ± 0.01	0.53 ± 0.04 **	0.35 ± 0.01	0.31 ± 0.03
Albumin (mg/dL)	71.72 ± 3.8	153.71 ± 2.8	195.27 ± 8.3 **	162.31 ± 9.2	142.85 ± 5.4

**Table 2:** Effects of DEP exposure and RA treatment on serum lipid profiles and inflammatory markers in mice. Serum levels of total cholesterol (T-Cho), triglycerides (TGs), HDL cholesterol (HDL-Cho), LDL cholesterol (LDL-Cho), atherogenic index, and high-sensitivity C-reactive protein (hs-CRP) were measured in each experimental group. Data are presented as mean ± SEM. * *p* < 0.05, ** *p* < 0.01, *** *p* < 0.001 vs. control; ## *p* < 0.01, ### *p* < 0.001 vs. ApoE−/−; $$ *p* < 0.01, $$$ *p* < 0.001 vs. ApoE−/− PM.

	Control	ApoE−/−	PM	Tret-Low	Tret-High
T-Cho (mg/dL)	62.68 ± 2.01	165.74 ± 3.65 **	176.47 ± 2.47 ##	91.85 ± 2.61 $$	85.36 ± 2.44 $$
TGs (mg/dL)	75.84 ± 1.26	110.22 ± 2.87 **	118.14 ± 3.54 ##	88.67 ± 2.88 $$	82.65 ± 1.47 $$
HDL-Cho (mg/dL)	53.89 ± 3.73	12.86 ± 0.60 **	5.06 ± 1.84 ###	20.79 ± 4.93 $$	21.69 ± 0.73 $$
LDL-Cho (mg/dL)	35.11 ± 6.23	102.11 ± 5.28 ***	119.66 ± 0.88 ###	47.66 ± 3.65 $$$	54.00 ± 6.73 $$$
Atherogenic index	0.16 ± 0.001	11.89 ± 0.002	33.88 ± 0.015 ###	3.42 ± 0.026 $$$	2.94 ± 0.017 $$$
hs-CRP (ng/dL)	4.28 ± 0.72	9.27 ± 0.85 *	11.01 ± 0.66 ##	9.71 ± 0.32	6.37 ± 0.22 $$

## Data Availability

Data is contained within the article or Supplementary Materials.

## Supplementary Materials

The following supporting information can be downloaded at: https://www.mdpi.com/article/10.3390/nu18213680/s1, Supplementary Material S1: Identification of target genes in human lung tissue of COPD patients (GSE106986); Supplementary Material S2: Retinoic Acid was identified as a candidate compound through L1000CDS^2^; Supplementary Material S3: Gene network visualization of lung- and cardiovascular disorder-related genes using various keywords; Supplementary Material S4: Assessment of eNOS connectivity in the lung- and cardiovascular disorder-related gene network; Table S1: List of upregulated and downregulated genes involved in the progression of pulmonary injury from human lung tissues and their associated Agilent probe identifiers used for initial L1000CDS^2^; Table S2: L1000CDS^2^ permuted results showing compounds with significant positive correlation to genes involved in the progression of pulmonary injury from human lung tissues. Table S3: CTD queries for identifying genes associated with pulmonary and cardiovascular disorders.

## Abbreviations

ApoE−/−: apolipoprotein E-deficient; BH2, dihydrobiopterin; BH4, tetrahydrobiopterin; COPD, chronic obstructive pulmonary disease; DEGs, differentially expressed genes; DEP, diesel exhaust particles; eNOS, endothelial nitric oxide synthase; GTPCH-1, GTP cyclohydrolase-1; HUVECs, human umbilical vein endothelial cells; NO, nitric oxide; RA, retinoic acid.
