# Supplementary material for: Retinoic Acid Alleviates Accelerated Atherosclerosis in Diesel Exhaust–Derived PM2.5–Exposed ApoE−/− Mice by Enhancing eNOS–NO Coupling
Source: Nutrients. 2026 Nov 6;18(21):3680. doi: 10.3390/nu18213680 (PMC13765351; doi:10.3390/nu18213680)
Supplement: Supplementary file 1 [file nutrients-18-03680-s001.zip › nutrients-4599506-supplementary-Tables.pdf]

Table S1. List of up-regulated and down-regulated genes involved in the progression of pulmonary injury from human lung tissues and their associated Agilent probe identifiers used for initial L1000CDS².

No.	Up-regulated probes		Down-regulated probes	
	Agilent ID	Gene symbol	Agilent ID	Gene symbol
1	A_23_P259314	RPS4Y1	A_33_P3234814	-
2	A_23_P324384	RPS4Y2	A_23_P170888	DPP6
3	A_23_P148088	FGG	A_33_P3347503	-
4	A_33_P3224331	DDX3Y	A_33_P3226420	RSAD2
5	A_24_P146683	MSMB	A_33_P3243405	GPR182
6	A_23_P154784	BPIFB1	A_33_P3229390	-
7	A_23_P73848	NCRNA00185	A_33_P3230498	-
8	A_33_P3217700	TTTY15; USP9Y	A_33_P3424057	PEG3
9	A_33_P3260223	TXLNGY	A_32_P381593	SLC26A7
10	A_33_P3241511	SERPIND1	A_23_P85218	SOX3
11	A_23_P30126	FGFBP1	A_33_P3302275	CXorf66
12	A_23_P137238	KDM5D	A_33_P3376131	NT5DC4
13	A_33_P3245228	BPIFA1	A_32_P8221	GRM8
14	A_23_P39550	TMEM163	A_23_P369343	KLK8
15	A_23_P126278	CHIT1	A_33_P3321919	SPATA45
16	A_24_P271696	XAGE1A	A_33_P3327519	SNORA74A
17	A_33_P3228977	UTY	A_33_P3332414	ABCB1
18	A_33_P3388501	CHIT1	A_33_P3367491	-
19	A_23_P153616	MADCAM1	A_33_P3300430	FBN3
20	A_23_P358709	AHRR	A_33_P3355266	TINAGL1
21	A_24_P227141	ELF5	A_33_P3336223	-
22	A_23_P364792	TXLNG2P	A_24_P391574	LDLRAD3
23	A_23_P150343	SLN	A_33_P3316770	SLCO1A2
24	A_23_P62309	AGTR2	A_32_P75902	C16orf73
25	A_23_P321949	PLA2G2A	A_24_P128442	TBX15
26	A_32_P86578	LOC389023	A_33_P3432135	EPHA1-AS1
27	A_23_P206077	AEN	A_23_P153480	KLK5
28	A_23_P369899	TMEM158	A_32_P111996	MGC39584
29	A_33_P3308232	-	A_33_P3405911	TSIX
30	A_23_P323751	FAM83D	A_23_P25150	HOXC9
31	A_24_P12397	TREM2	A_33_P3387656	-
32	A_24_P153324		A_33_P3291976	TERF1
33	A_24_P264943	COMP	A_33_P3232173	-
34	A_23_P167159	SCRG1	A_23_P28012	ZNF682
35	A_23_P93141	GSTA5	A_33_P3366296	LINC01551
36	A_23_P55270	CCL18	A_33_P3745164	CADM2
37	A_23_P96658	TXLNG2P	A_24_P18802	VPS18
38	A_33_P3370019	-	A_32_P211117	HOXD3
39	A_33_P3424754	-	A_33_P3576317	-
40	A_32_P33114	KLB	A_33_P3293913	BICC1
41	A_23_P160004	UTY	A_33_P3347417	SPEN

42	A_33_P3284253	GYG2	A_33_P3320808	NDUFS7
43	A_33_P3365437	LHX9	A_33_P3213832	-
44	A_23_P7313	SPP1	A_24_P941736	ACSBG1
45	A_24_P125335	CCL13	A_23_P119448	PPP6R1
46	A_23_P71037	IL6	A_33_P3315263	KRT79
47	A_33_P3281435	-	A_23_P81507	FAT2
48	A_23_P17065	CCL20	A_23_P39056	KLK7
49	A_23_P375372	FGA	A_23_P155463	LRRC2
50	A_23_P169437	LCN2	A_33_P3236416	GPR179
51	A_23_P331049	DPYSL4	A_24_P131589	CD86
52	A_33_P3284247	-	A_24_P932736	HMBOX1
53	A_23_P112482	AQP3	A_33_P3341686	XIST
54	A_24_P277367	CXCL5		
55	A_24_P297078	C20orf3		
56	A_23_P6119	SEC23B		
57	A_24_P413669	PFKFB2		
58	A_23_P5211	MUC16		
59	A_32_P80245	ZFP57		
60	A_33_P3304696	-		
61	A_32_P107029	NAPSA		
62	A_32_P62963	KRT16P2		
63	A_32_P70315	TIMP4		
64	A_24_P6370	C1orf110		
65	A_23_P12746	MRC1		
66	A_23_P55632	SERPINB3		
67	A_24_P158089	SERPINE1		
68	A_32_P149435	PTCHD1		
69	A_32_P190049	LRRC58		
70	A_33_P3408918	SAA2		
71	A_23_P502413	SERPINB4		
72	A_24_P935009	CRKL		
73	A_24_P140608	HBEGF		
74	A_23_P80974	TDO2		
75	A_33_P3319870	GREM1		
76	A_23_P364324	ABCA13		
77	A_24_P237511	EIF1AY		
78	A_33_P3253598	-		
79	A_23_P143526	S100B		
80	A_23_P146512	GOLM1		
81	A_23_P92202	GMPPB		
82	A_33_P3367850	CHRM4		
83	A_24_P348861	TTTY15		
84	A_24_P208825	MUC4		
85	A_23_P218047	KRT5		
86	A_23_P126677	DNASE2B		
87	A_23_P130961	ELANE		
88	A_23_P204304	PTPRO		

89	A_33_P3233040	SERPINB11		
90	A_33_P3725324	TTTY15; USP9Y		
91	A_33_P3290343	CYP1B1		
92	A_23_P8702	PIP		
93	A_32_P163147	VSIG1		
94	A_24_P183150	CXCL3		
95	A_24_P406334	STEAP1		
96	A_33_P3281444	-		
97	A_23_P168761	PTPRZ1		
98	A_23_P24129	DKK1		
99	A_23_P17821	PLA2G3		
100	A_33_P3249046	CLDN2		
101	A_23_P214267	GPR110		

Table S2. L1000CDS² permuted results showing compounds with significant positive correlation to genes involved in the progression of pulmonary injury from human lung tissues.

Rank	Perturbation	enrichment	p
1	vorinostat	-0.884	0.00042
2	geldanamycin	0.984	0.00052
3	Retinoic acid	-0.824	0.00185
4	Ro 31-8220 mesylate	-0.822	0.00189
5	trichostatin A	0.816	0.00209
6	G5797	0.749	0.00234
7	belinostat	-0.798	0.00332
8	selumetinib	-0.655	0.00469
9	nintedanib	-0.691	0.00637
10	EPIRUBICIN HYDROCHLORIDE	-0.762	0.00660
11	PX12	-0.941	0.00742
12	CLONIDINE HYDROCHLORIDE	0.841	0.00793
13	MLN4924	-0.731	0.01056
14	BML-259	0.660	0.01204

Table S3. CTD queries for identifying genes associated with pulmonary and cardiovascular disorders.

No.	Symbol	Entrez Gene Name	Location	Type(s)
1	ADORA2B	adenosine A2b receptor	Plasma Membrane	G-protein coupled receptor
2	ANGPT1	angiopoietin 1	Extracellular Space	growth factor
3	ARG1	arginase 1	Cytoplasm	enzyme
4	ASH2L	ASH2 like, histone lysine methyltransferase complex subunit	Nucleus	transcription regulator
5	BIRC5	baculoviral IAP repeat containing 5	Cytoplasm	other
6	bleomycin	--	Other	biologic drug
7	C/EBP	--	Cytoplasm	group
8	CAV1	caveolin 1	Plasma Membrane	transmembrane receptor
9	CSF2	colony stimulating factor 2	Extracellular Space	cytokine
10	CXCR4	C-X-C motif chemokine receptor 4	Plasma Membrane	G-protein coupled receptor
11	dasatinib	--	Other	chemical drug
12	EPAS1	endothelial PAS domain protein 1	Nucleus	transcription regulator
13	etretinate	--	Other	chemical drug
14	F2RL2	coagulation factor II thrombin receptor like 2	Plasma Membrane	G-protein coupled receptor
15	F2RL3	F2R like thrombin or trypsin receptor 3	Plasma Membrane	G-protein coupled receptor
16	FCGR2A	Fc fragment of IgG receptor IIa	Plasma Membrane	transmembrane receptor
17	FLT3LG	fms related receptor tyrosine kinase 3 ligand	Extracellular Space	cytokine
18	FOSL2	FOS like 2, AP-1 transcription factor subunit	Nucleus	transcription regulator
19	IL2	interleukin 2	Extracellular Space	cytokine
20	IL18	interleukin 18	Extracellular Space	cytokine
21	L-propargylglycine	--	Other	chemical reagent
22	lipopolysaccharide	--	Other	chemical drug
23	LTA	lymphotoxin alpha	Extracellular Space	cytokine
24	monocrotaline	--	Other	chemical toxicant
25	MRTFA	myocardin related transcription factor A	Nucleus	transcription regulator
26	N-acetyl-L-cysteine	--	Other	chemical drug
27	NOS2	nitric oxide synthase 2	Cytoplasm	enzyme
28	NOS3	nitric oxide synthase 3	Cytoplasm	enzyme
29	particulate matter	--	Other	chemical reagent
30	PDCD4	programmed cell death 4	Nucleus	other

31	PFKFB3	6-phosphofructo-2-kinase/fructose-2,6-biphosphatase 3	Cytoplasm	kinase
32	PRF1	perforin 1	Cytoplasm	transporter
33	PTGDR2	prostaglandin D2 receptor 2	Plasma Membrane	G-protein coupled receptor
34	REGN421	--	Other	biologic drug
35	RPTOR	regulatory associated protein of MTOR complex 1	Cytoplasm	other
36	semaxinib	--	Other	chemical drug
37	SLC6A4	solute carrier family 6 member 4	Plasma Membrane	transporter
38	SLC9A1	solute carrier family 9 member A1	Plasma Membrane	ion channel
39	TNFSF4	TNF superfamily member 4	Extracellular Space	cytokine
40	TNFSF10	TNF superfamily member 10	Extracellular Space	cytokine
41	WDR5	WD repeat domain 5	Nucleus	transcription regulator
